# Developing Guidance on Implementing Volunteer-Led Intradialytic Arts Activities in Haemodialysis Units

**DOI:** 10.3390/healthcare9111506

**Published:** 2021-11-05

**Authors:** Anna Wilson, Claire Carswell, Helen Noble

**Affiliations:** School of Nursing and Midwifery, Queen’s University Belfast, Belfast BT7 1NN, UK; anna.wilson@qub.ac.uk (A.W.); c.carswell@qub.ac.uk (C.C.)

The Renal Arts Group (RAG) at Queen’s University Belfast was formed in 2016 as a collaboration between patients with kidney disease, carers, clinicians, academics and artists to develop a programme of research with the ultimate aim of improving the physical and psychological quality of life of those living with kidney disease through the medium of the arts [1]. The group aims to give a voice to patients living with kidney disease, increase public engagement and improve social impact through interdisciplinary collaboration between academics, researchers, healthcare staff, policy makers and service users. In 2019 RAG Co-Chair Dr Helen Noble, along with RAG members Dr Claire Carswell and Anna Wilson, undertook an initiative to develop guidance for implementing volunteer-led arts activities for patients undergoing haemodialysis.

The initiative builds upon Dr Carswell’s PhD study [2,3], which consisted of a pilot feasibility trial of an arts-based intervention for patients receiving haemodialysis. The study aimed to address quality of life and mental health of patients who have end-stage kidney disease (ESKD) and are receiving haemodialysis. Haemodialysis requires patients to attend hospital three times a week for a period of four hours each visit, during which patients are connected to a dialysis machine and restricted to a hospital bed [4]. The kidney disease plus the amount of time dedicated to this treatment and the experience of existential boredom associated with the time spent on the machine [5], can lead to anxiety [6], depression [7] and poor quality of life [8]. There is growing evidence for the role that the arts can play in improving health and wellbeing, including benefits for mental health and the management of chronic, complex and long term conditions [9], and the Creative Health: The Arts for Health and Wellbeing report recommends an increase in collaboration between leaders from the arts, health and social care sectors, service users and academics to advance good practice and inform policy [10]. The pilot trial found that art can be safely implemented in this clinical setting, that the intervention was highly acceptable and that it improved the treatment experience for both patients and staff. Patients reported positive experience including increased self-esteem, development of a sense of purpose, feeling happy and increased social interaction, and healthcare professionals reported to have experienced improved communication with patients during the study [3].

Traditionally, arts in health programmes are facilitated by professional artists who are employed as an artist-in-residence within a hospital or healthcare trust, who often undertake larger collaborative projects within different areas of the hospital or trust and have limited time to dedicate to each units’ specific needs. In order to facilitate undertaking a programme of arts activities with individual patients, the research team, in consultation with the Northern Health and Social Care Trust (NHSCT), took the decision that trained volunteers would be well placed to support the work of the artist-in-residence, whilst being able to spend longer periods of time with individual patients to focus on skills development and ensure sustainability and engagement of the arts intervention.

The research team undertook regular consultations with members of the project advisory group, which included volunteering and healthcare professionals from the NHSTC, working closely with the Community Wellbeing Manager and Volunteer Co-ordinator, to inform the development of a guidance document outlining the implementation of a volunteer-led arts programme that could be replicated in renal units across Northern Ireland and the UK. Working collaboratively allowed the team to identify key considerations required for the guidance document including training requirements for potential volunteers, a detailed description of the volunteer role, recruitment strategies for the post and to establish governance and standards considerations for implementation. Careful consideration was taken regarding the skills required for the role, and the group identified that, while an interest in art would be helpful for the role, it would be most important for the volunteer to have good communication and interpersonal skills and be able to provide a supportive learning experience for the patient.

The initiative originally planned to develop guidance for direct one-to-one bedside facilitation of arts activities; however, the restrictions imposed by the pandemic, including enhanced infection control protocols within hospitals and risk management strategies employed by health and social care trusts [11] meant that a flexible and agile approach had to be considered. The guidelines now reflect the need for virtual volunteers, which will allow support for the patients to continue when non-clinical staff are unable to access the renal unit. Virtual volunteers can offer one-to-one facilitation over telephone or video calls to provide encouragement and assistance while maintaining the momentum of the arts activity programme during periods of restricted access to the unit. While restrictions due to the pandemic have significantly relaxed in the UK, patients with ESKD remain vulnerable to the threat of COVID-19 [12] and the need for virtual volunteers may continue for the foreseeable future.

As part of the initiative, a virtual workshop was held with healthcare professionals including renal healthcare professionals, along with patients, artists and representatives from voluntary arts and health organisations and renal charities, to further refine the guidance document and ensure that all stakeholder requirements were addressed within the proposed guidelines. Additionally, a focus group was held with key representatives from the NHSCT volunteering, wellbeing and clinical teams along with Patient Support and Advocacy Officers from Kidney Care UK to gather feedback and inform the dissemination strategy.

The guidelines have been disseminated online and are supported by an informative and engaging animation and infographic developed in partnership with research communications agency Science Animated [13]. The associated documents and media are available for download from the Renal Arts Group website www.qub.ac.uk/sites/renal-arts-group/Research/VolunteerGuidelines/ [14].
